# CXCL5 Has Potential to Be a Marker for Hepatocellular Carcinoma Prognosis and Was Correlating With Immune Infiltrates

**DOI:** 10.3389/fonc.2021.637023

**Published:** 2021-03-31

**Authors:** Yuan Nie, Mei-chun Jiang, Cong Liu, Qi Liu, Xuan Zhu

**Affiliations:** Department of Gastroenterology, The First Affiliated Hospital of Nanchang University, Nanchang, China

**Keywords:** CXCL5, hepatocellular carcinoma, tumor microenvironment, immune infiltration, prognosis

## Abstract

**Backgrounds:**

Tumor microenvironment (TME) plays a crucial role in the initiation and progression of Hepatocellular Carcinoma (HCC), especially immune infiltrates. However, there is still a challenge in understanding the modulation of the immune and stromal components in TME, especially TME related genes.

**Methods:**

The proportion of tumor-infiltrating immune cells (TICs) and the immune and stromal scores in 374 HCC patients from The Cancer Genome Atlas (TCGA) database were determined using CIBERSORT and ESTIMATE computational methods. The final screened genes were confirmed by the PPI network and univariate Cox regression of the differentially expressed genes based on different immune or stromal scores. The correlation between the expression levels of the final gene interactions and the clinical characteristics was based on TCGA database and local hospital data. Gene set enrichment analysis (GSEA) and the effect of CXCL5 expression on TICs were conducted.

**Results:**

There were correlations between the expression of CXCL5 and survival of HCC patients and TMN classification both in TCGA database and local hospital data. The immune-related activities were enriched in the high-expression group; however, the metabolic pathways were enriched in the low-expression group. The result of CIBERSORT analyzing had indicated that CXCL5 expression were correlated with the proportion of NK cells activated, macrophages M0, Mast cells resting, Neutrophils.

**Conclusions:**

CXCL5 was a potential prognostic marker for HCC and provides clues regarding immune infiltrates, which offers extra insight for therapeutics of HCC, however, more independent cohorts and functional experiments of CXCL5 are warranted.

## Introduction

Liver cancer is a typical inflammation driven tumor and often develops from chronic hepatitis and cirrhosis (1). Hepatocellular carcinoma (HCC) accounts for 75–85% of primary liver cancers, ranking sixth among the most common cancers in the world, and fourth among cancer-related deaths. Eighty-five percent of all HCC patients occurs in poor or developing countries, especially in East Asia and Africa (2). The main treatment methods include surgery, liver transplantation, local ablation, molecular targeted therapy, and systemic chemotherapy, had limitation on the improvement of patient’s survival and imposes a heavy burden on health-care costs. It is urgently needed to explore the carcinogenesis and therapeutics of HCC (3).

Increasing evidence demonstrated the importance of the tumor microenvironment (TME) in the tumor development, especially in HCC (4). As an inflammatory tumor, the immunosuppressive microenvironment of HCC can promote immune tolerance through a variety of mechanisms. Immunotherapy that activates tumor specific immune response brings new hope for the treatment of HCC. The microenvironment of HCC is mainly composed of tumor associated macrophages, tumor associated neutrophils and myeloid-derived suppressor cells (MDSCs), tumor associated fibroblasts, tumor infiltrating lymphocytes and other cellular components, as well as extracellular matrix, cytokines, and other non-cellular components. Previous studies showed that the tumor-infiltrating immune cells (TICs) in TME plays an important role in development of HCC and served as a predicting parameter for prognosis. For example, Kupffer cells play an important role in inhibitory microenvironment by producing anti-inflammatory molecules such as TGF -β, IL-10, and prostaglandin E2 (PGE2) (5). Interferon-γ (IFN-γ) derived from Natural killer (NK) cells promotes HCC through the epithelial cell adhesion Molecule-Epithelial-to-Mesenchymal Transition (EMT) axis in Hepatitis B virus (HBV) transgenic mice. A previous study had indicated that neutrophil to lymphocyte ratio and platelet to lymphocyte ratio as prognostic predictors for HCC with various treatments (6). Immune tolerance is one of the main causes of the adverse consequences of high mortality, poor therapeutic effect, and poor prognosis of HCC (7). Immune cells in tumor microenvironment together with cancer cells and extracellular matrix, thus inhibiting the antitumor activity of immune cells and playing an important role in promoting of HCC. Therefore, the analysis of TICs of HCC is helpful to study the pathogenesis of HCC.

Transcriptome-sequencing patterns followed by functional genomics analysis have shed light on the roles of different types of cells during TME modulation. In this paper, we calculated the ratio of tic and immune/stromal components of HCC patients in The Cancer Genome Atlas (TCGA) database by using ESTIMATE and CIBERSORT, and determined that C-X-C Motif Chemokine Ligand 5 (CXCL5) is a predictive biomarker. CXCL5, also known as human epithelial neutrophil activating peptide (ENA 78), is a member of angiogenic CXC chemokine. CXCL5 is secreted by epithelial cells, endothelial cells, immune cells, etc. and recognized and combined with the G protein coupled receptor CXCR2 (8). It can recognize and bind to CXCR2, and perform many cellular functions including adhesion, invasion, and diffusion through autocrine or non-autocrine pathways, thus affecting the growth, proliferation, metastasis, and invasion of tumors. CXCL5 is secreted not only by neutrophils, monocytes, and megaphone immune cells, but also by non-immune cells such as epithelial cells, endothelial cells, and fibroblasts. As an inflammatory mediator, CXCL5 has a strong chemotactic effect on neutrophils and can activate neutrophils, suggesting that CXCL5 might play a role in TME (9). Hence, we examined the differentially expressed genes (DEGs) generated by comparison between immune components and stromal components in HCC samples and revealed that the CXCL5 might be a potential indicator for the alteration of TME status in HCC.

## Materials and Methods

### Raw Data

Transcriptome RNA-seq data of 424 HCC samples (normal samples, 50 cases; tumor samples, 374 cases) and the corresponding clinical data were downloaded from TCGA database (https://portal.gdc.cancer.gov/). At the same time, the blood sample of HCC patients in hospital were collected in this study. Refusal to give consent, cerebrovascular disease, cardiovascular disease, hematologic disorders, renal failure, combined other cancer, and correspond treatment were exclusion criteria. The study protocol was approved by the institutional ethics committee of First Affiliated Hospital of Nanchang University (No. 2017-0106). Written informed consent was obtained from all the study participants.

### Bioinformatics Analysis

The ratio of immune-stromal component in TME was calculated by Using the Feat estimation algorithm in R language version 3.5.1, which expressed in three scoring forms: Immune Score, Stromal Score, and ESTIMATE Score. According the median of the Immune score, Stromal Score, and ESTIMATE Score, tumor samples were labeled as high or low. The differential expression genes (DEGs) was generated by comparing high score samples with low score samples in package limma. DEGs with fold change larger than 1 after transformation of log_2_(high-score group/low-score group) and false discovery rate (FDR) < 0.05 were considered significant.

GO and KEGG enrichment analyses were performed by packages clusterProfiler, enrichplot, and ggplot2 of R language. Only terms with both P value and q-value < 0.05 were considered significantly enriched. PPI network was constructed by STRING database, followed by reconstruction with Cystoscope of version 3.6.1. Nodes with confidence of interactive relationship larger than 0.95 were used for building network.

Hallmark and C7 gene sets v 6.2 collections were downloaded from Molecular Signatures Database as the target sets with which GSEA performed using the software GESA 3.0. The whole transcriptome of all tumor samples was used for GSEA, and only gene sets with NOM p < 0.05 and

FDR q < 0.05 were considered as significant. CIBERSORT computational method was applied for estimating the TIC abundance profile in all tumor samples, and only tumor samples with P < 0.05 were selected for the following analysis.

### Definitions

Patients with chronic HBV infection were confirmed by the detection of Hepatitis B surface antigen (HBsAg) positivity for more than 6 months. The liver cirrhosis was diagnosed by the presence of ascites, hepatic encephalopathy (HE), Hepatorenal syndrome (HRS), and/or variceal bleeding at the time of the study. The diagnosis of HCC and TMN classification was mainly based on pathological and clinical characteristics.

### ELISA and Real-Time Quantitative PCR

Peripheral venous blood was collected through the elbow vein, centrifuged at 3,000 r/min for 10 min, and the supernatant was stored in −80°C refrigerator. Serum CXCL5 level was determined by the standard photometric method using the ELISA kit (R&D company, USA). For real-time PCR analysis, PCR was performed with a reaction mixture containing cDNA template, primers, and TB Green™ Fast qPCR Mix (TaKaRa) in a Step One Plus Real-Time PCR System (Thermo Fisher Scientific).

The primers of CXCL5 were 5’-CCGCTGCTGTGTTGAGAG-3’ and 5’-TCTGCTGAAGACTGGGAAAC-3’.

### Statistical Analysis

Statistical analyses were performed using SPSS software version 16.0 (SPSS Inc., Chicago, IL, USA) and R 3.62. Continuous and categorical variables were initially described as median [interquartile range (IQR)] and frequency [percentage (%)]. Univariate Cox regression was used to completed by package survival of R language and the top 18 genes ordered by p value from small to large in univariate Cox were shown in the plot. Survival analysis was completed by the survival and survminer package of R language. Kaplan–Meier (K-M) method was used to plot the survival curve, and log rank as the statistical significance test. Heatmaps of DEGs were produced by package heatmap of R language. P < 0.05 was considered significant.

## Results

**Figure 1:**
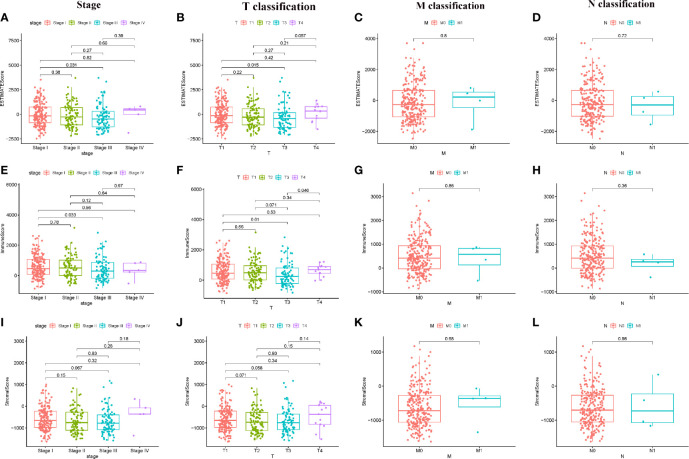
Correlation of ESTIMATE Score, Immune Score, and Stromal Score with clinical characteristics. **(A–D)** Distribution of ESTIMATE Score in different stage and TMN classification; **(E–H)** Distribution of Immune Score in different stage and TMN classification; **(I–L)** Distribution of Stromal Score in different stage and TMN classification.

Analysis process of this study was shown in **Figure 1**. The transcriptome RNA-seq data of 424 cases were downloaded from TCGA database followed by calculating with CIBERSORT and ESTIMATE algorithms. Protein-protein interaction (PPI) network was constructed by using DEGs shared by Immune score and Stromal score, and Univariate Cox regression analysis was conducted. Intersection analysis was performed using the core nodes in PPI network and the top significant factors obtained from the analysis of univariate Cox regression. We focused on CXCL5 for the subsequent series of analysis, including survival and clinicopathological characteristics correlation analysis, Cox regression, GSEA, and correlation with TICs.

### Scores Were Associated With the Clinical Characteristics of HCC Patients

In order to determine the relationship between Immune score, Stromal score, ESTIMATE Score with clinical characteristics, the clinical information of HCC patients from TCGA were collected. The analyzing result were shown in **Figure 1**. The ESTIMATE Score of stage III is significantly lower than that of stage I (P = 0.031); the ESTIMATE Score of T3 classification of TMN stages is significantly lower than that of T1 classification (P = 0.015). The Immune Score of stage III is significantly lower than that of stage I (P = 0.033); the Immune Score of T3 classification of TMN stages is significantly lower than that of T1 classification (P = 0.010). There are no significant different in comparing of Stromal score (P > 0.05). These results suggested that TME was associated with the progress of HCC, especially immune related tumor microenvironment.

### DEGs Between Lower Immune Score, Stromal Score and Higher Immune Score, Stromal Score

**Figure 2:**
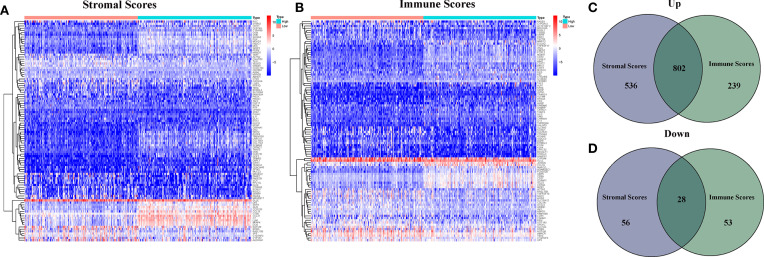
Heatmaps and Venn plots for DEGs. **(A)** Heatmap for DEGs generated by comparison of the high score group *vs* the low score group in Stromal Score; **(B)** Heatmap for DEGs generated by comparison of the high score group *vs* the low score group in Immune Score; **(C, D)** Venn plots showing common up-regulated and down-regulated DEGs shared by Immune Score and Stromal Score.

In order to determine the different of gene expression, the gene expression of high and low score samples were compared and analyzed. As shown in **Figure 2**, Compared to the median, the total 1,422 DEGs were obtained from Stromal Score (samples with high score *vs.* low score). Similarly, 1,122 DEGs were obtained from Immune Score. The intersection analysis displayed by Venn plot showed a total of 802 up-regulated genes sharing by high score both in Immune Score and Stromal Score and 28 down-regulated genes sharing by low score as well.

### Enrichment Analysis of GO and KEGG

**Figure 3:**
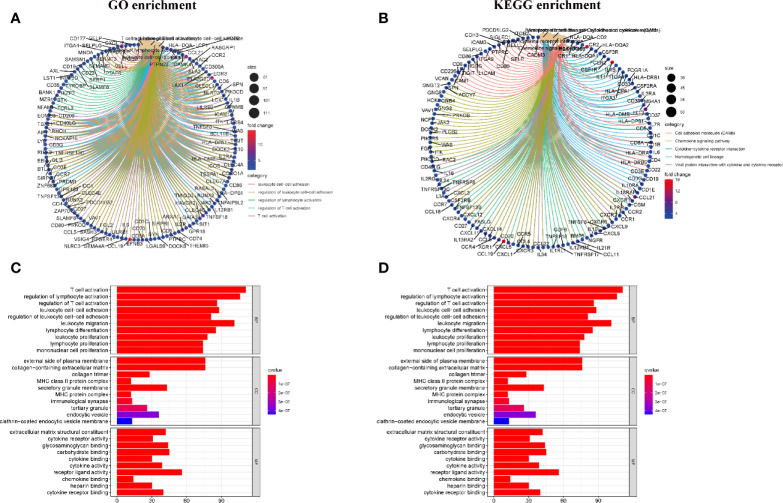
Enrichment analysis of GO and KEGG for DEGs. **(A, C)** GO enrichment analysis for 830 DEGs; **(B, D)** KEGG enrichment analysis for 830 DEGs.

As shown in **Figure 3**, the results of gene ontology (GO) enrichment analysis indicated that the DEGs almost mapped to the immune-related GO terms, such as T cell activation, regulation of lymphocyte activation (**Figures 3A, C**). The Kyoto Encyclopedia of Genes and Genomes (KEGG) enrichment analysis also displayed the enrichment of T cell activation, regulation of lymphocyte activation (**Figures 3B, D**). Therefore, the overall function of DEGs seems to map to immune related activities, which indicates that the involvement of immune factors is a major feature of TME in HCC.

### Intersection Analysis of PPI Network and Univariate COX Regression

**Figure 4:**
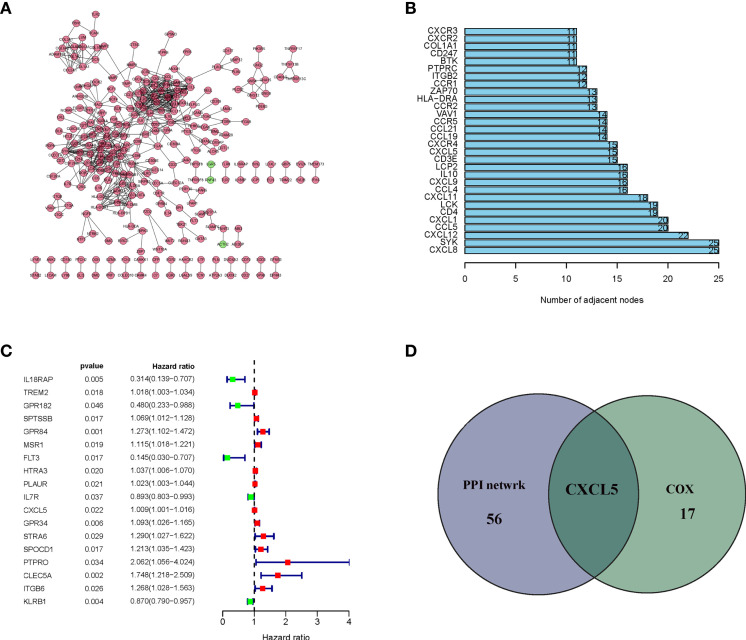
Protein–protein interaction network and univariate cox. **(A)** Interaction network constructed with the nodes with interaction confidence value > 0.95; **(B)** The top 30 genes ordered by the number of nodes; **(C)** Univariate cox regression analysis with 830 DEGs, listing the top significant factors with P < 0.005; **(D)** Venn plot showing the common factors shared by leading 30 nodes in PPI and top significant factors in univariate cox.

In order to further explore the underlying mechanism, PPI network based on String database by using the Cytoscape software was conducted. The interactions are shown in **Figure 4A**, and the bar plots were represented for the top 30 genes ranked by the number of nodes (**Figure 4B**). Univariate Cox regression analysis was used to determine the significant factors affecting the survival of HCC patients (**Figure 4C**). And then, the intersection analysis between the leading nodes in PPI network and the top 16 factors ranked by the p-value of univariate Cox regression was carried out, and only one factor, CXCL5, was overlapping from the above analyses (**Figure 4D**).

### The Correlation of CXCL5 With Clinical Characteristics of HCC Patients in TCGA

**Figure 5:**
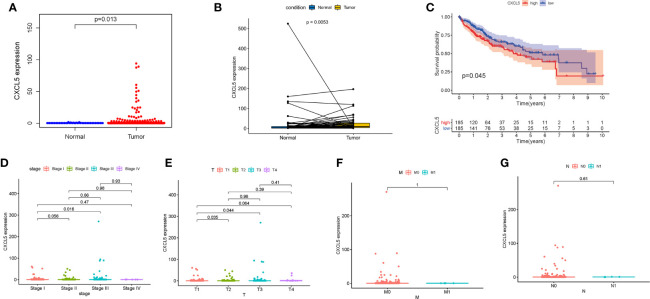
The differentiated expression of CXCL5 and correlation with survival and clinical characteristics. **(A)** Differentiated expression of CXCL5 in the normal and tumor sample; **(B)** Paired differentiation analysis for expression of CXCL5 in the normal and tumor sample deriving from the same one patient; **(C)** Survival analysis for HCC patients with different CXCL5 expression; **(D–G)** The correlation of CXCL5 expression with clinical characteristics.

In comparing of CXCL5 gene expression, the CXCL5 expression of normal patients was significantly lower than that of HCC patients (**Figure 5A**). According the gene expression of CXCL5, all HCC samples were grouped into high-expression group and low-expression group. The survival analysis that HCC patients with lower expression had longer survival than that of higher expression (**Figure 5C**). In the paring analysis, the expression of CXCL5 in the tumor samples was significantly lower than that in the normal samples (**Figure 5B**). The above results clearly indicated that the expression of CXCL5 in TME was positive correlation with the prognosis of HCC patient, especially in stage and T classification (**Figures 5D–G**).

### The Correlation of CXCL5 With Clinical Characteristics of HCC Patients in This Hospital

**Figure 6:**
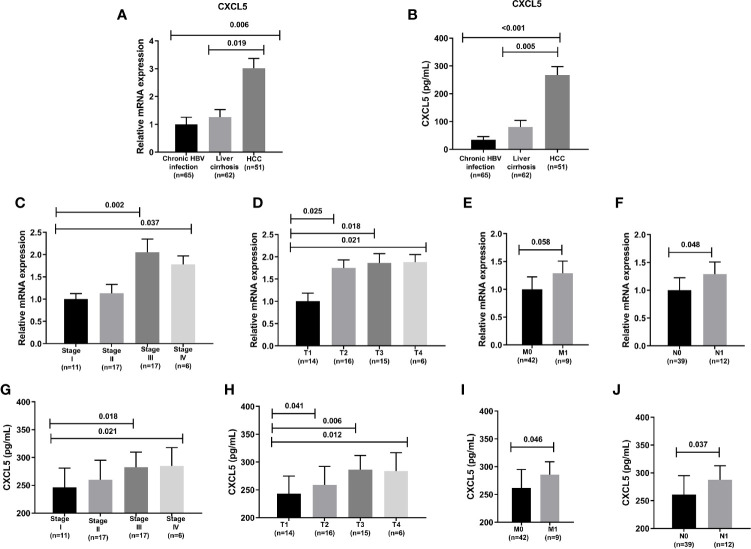
The CXCL5 level and correlation with clinical characteristics. **(A)** The relative mRNA expression of CXCL5 in chronic HBV infection, liver cirrhosis, HCC; **(B)** The CXCL5 level by ELISA in chronic HBV infection, liver cirrhosis, HCC; **(C–F)** The correlation of CXCL5 mRNA expression with clinical characteristics; **(G–J)** The correlation of CXCL5 level by ELISA with clinical characteristics.

There were 65 patients with Chronic HBV infection, 62 patients with liver cirrhosis, 52 patients with HCC of this hospital in this study. The relative mRNA expression of CXCL5 of HCC patients were higher than patients with liver cirrhosis, patients with Chronic HBV infection (**Figure 6A**), also the ELISA detected results of CXCL5 of HCC patients were higher than patients with liver cirrhosis, patients with Chronic HBV infection (**Figure 6B**). In 52 HCC patients, the relative mRNA expression of CXCL5 also is positive correction with the TMN classification (**Figures 6C–F**). Also, the same result of serum CXCL5 by ELISA was shown in **Figures 6G–J**.

### CXCL5 May Be a Potential Indicator of TME Modulation

**Figure 7:**
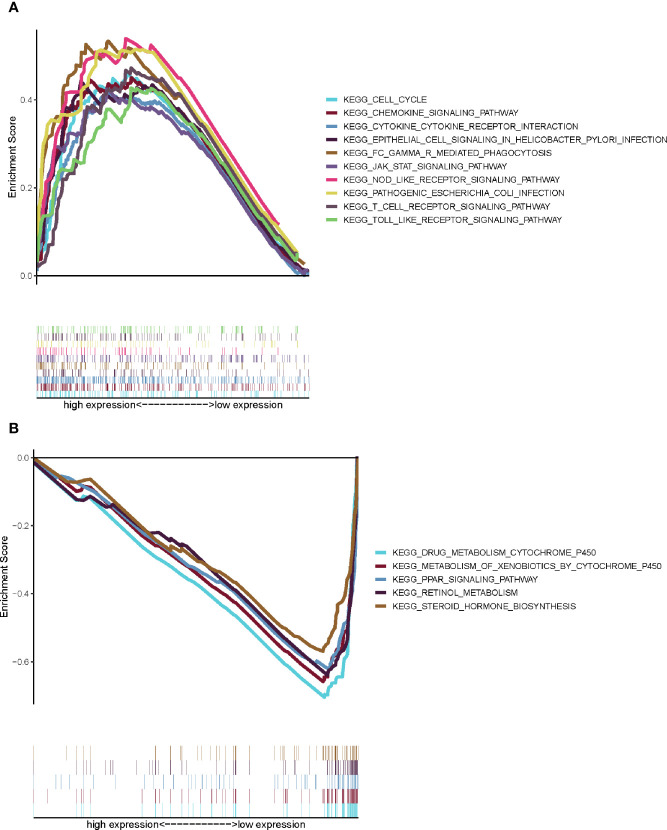
GSEA for samples with high CXCL5 expression and low expression. **(A)** GSEA for samples with high CXCL5 expression; **(B)** GSEA for samples with low CXCL5 expression.

Comparing with the median level of CXCL5 expression, GESA of CXCL5 in high- and low-expression groups was completed. As shown in **Figure 7A**, the genes in CXCL5 high-expression group were mainly enriched in immune-related activities, such as cell cycle, chemokine signaling, NOD like receptor. As shown in **Figure 7B**, the genes in CXCL5 high-expression group were mainly enriched in metabolism pathways, such as drug metabolism cytochrome, metabolism of xenobiotics. It is suggested that CXCL5 may be a potential indicator of TME status.

### Correlation of CXCL5 With the Proportion of TICs

**Figure 8:**
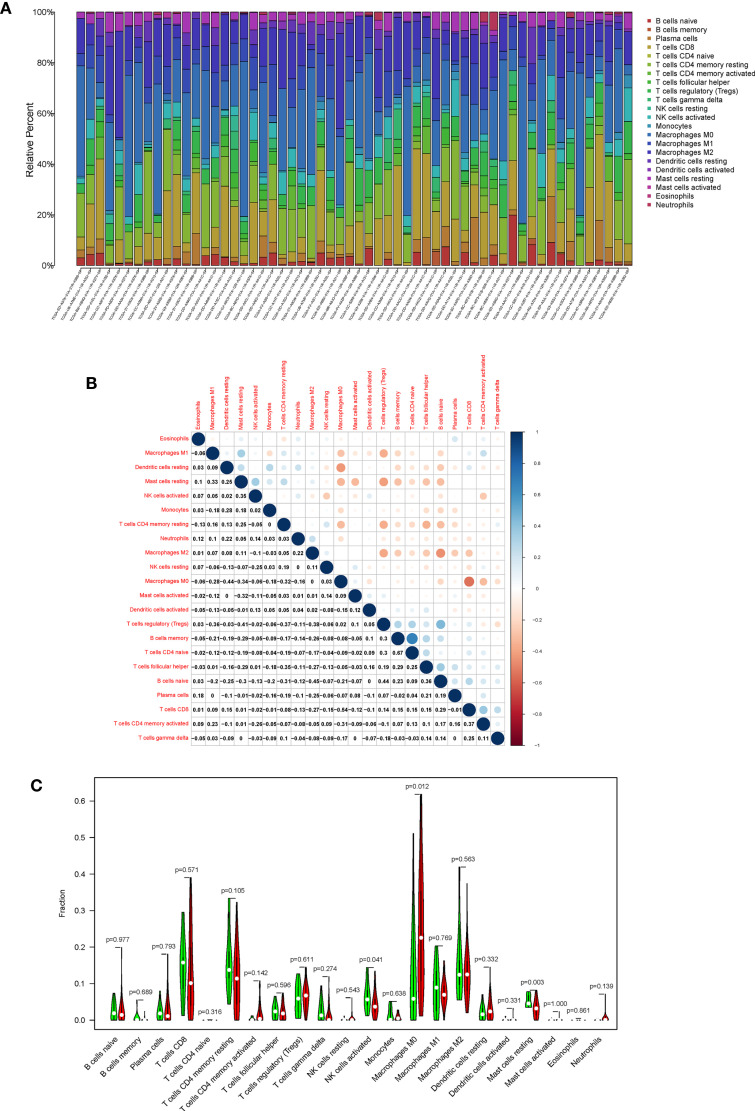
TIC profile in tumor samples and correlation analysis. **(A)** Barplot showing the proportion of 21 kinds of TICs in HCC tumor samples; **(B)** Heatmap showing the correlation between 21 kinds of TICs and numeric; **(C)** Violin plot showed the ratio differentiation of 21 kinds of immune cells between HCC samples with high CXCL5 expression and low CXCL5 expression.

In order to further confirm the correlation between CXCL5 expression and immune microenvironment, the proportion of tumor-infiltrating immune subsets was analyzed using CIBERSORT algorithm, and 21 kinds of immune cell profiles in HCC patients were completed (**Figures 8A, B**). The results showed that the NK cells activated, Mast cells resting of high-expression group of CXCL5 is significantly higher than that of low-expression group of CXCL5 (P = 0.041; P = 0.003); the macrophages M0 of high-expression group of CXCL5 is significantly lower than that of low-expression group of CXCL5 (P = 0.012) (**Figure 8C**). Also, there are significant correlation between CXCL5 expression and the proportion of NK cells activated, macrophages M0, Mast cells resting, Neutrophils (*r* = −0.31, *P* = 0.017; *r* = 0.37, *P* = 0.0041; *r* = −0.39, *P* = 0.0025; *r* = 0.35, *P* = 0.0077). These results further support the effect of CXCL5 expression on the immune activity of TME.

## Discussion

In the current study, we attempted to identify TME related genes that affect survival and TMN classification of HCC patients from TCGA database. Firstly, based on the DEGs between lower immune score, stromal score and higher immune score, stromal score, TME related genes was collected. Then, CXCL5 was identified to be involved by intersection analysis of PPI network and univariate cox regression. The gene expression of CXCL5 was significant correction with TMN classification and survival by TCGA database and local hospital data. Finally, CXCL5 might be an indicator of TME status in HCC patients.

TME plays a key role in tumorigenesis and development. As a congenital condition, TME promotes the occurrence and development of tumors (10). It is of great clinical significance to explore potential therapeutic targets based on TME remodeling and promote the transformation of TME from tumor friendly to tumor suppressor (11). A large number of studies have clarified the importance of TME in HCC. As expected, our transcriptome analysis of HCC data from TCGA database showed that the proportion of immune and stromal in TME was significantly correlated with HCC progression (such as invasion and metastasis) (**Figure 2**). These results highlight the value of TME in the development of HCC and provide a new perspective for the development of more effective treatment strategy. HCC as a typical inflammation-related tumor.

The microenvironment of liver cancer is mainly composed of tumor associated macrophages, tumor associated neutrophils, myeloid-derived suppressor cells (MDSCs), tumor associated fibroblasts, tumor infiltrating lymphocytes and dendritic cells (DCs), as well as non-cellular components such as extracellular matrix and cytokines (4). A study had indicated that the increase of CD4^+^CD25^+^ Treg cells in TME was related to tumor size, and these CD4^+^CD25^+^ Treg inhibited the immune response of DCs in HCC. NK cells are anti-tumor immune cells that play an indispensable role in tumor immune surveillance and tumor cell eradication. However, NK cell function is usually inhibited in tumor. miR-561-5p with high expression in HCC directly target to reduce the expression of CXCL3, reduce the infiltration of CXCR3^+^ NK cell subtypes in TME, promote the survival of cancer cells, and promote lung metastasis (12). Circ HURF derived from hepatoma cell exosomes can inhibit NK cell function, promote immune escape and resist PD-1 immunotherapy resistance through miR-449c-5p/TIM3 pathways. More evidence had indicated that macrophages promote tumor progression and metastasis (13). Osteopontin can stimulate macrophages to secrete colony stimulating factor (CSF1) through PI3K-Akt-p65 pathway, and then promote macrophage polarization by CSF1/CSF 1R pathway, up-regulate the expression of PD-L1 in HCC, create an inhibitory immune microenvironment, and induce immune escape of HCC. Also, the active mediator secreted by Mast cells in hepatocellular carcinoma tissue can make hepatic sinusoidal endothelial cells capillary, resulting in thickening of the basement membrane, and then forming new capillaries to increase the blood supply of tumor tissue, thereby promoting the proliferation and invasion of cancer cells (14).

The type, location, and density of immune cell infiltration in different tumor areas (i.e., tumor center and invasive margin) are evaluated respectively, which is called “immune score.” The immune score can not only reveal the immune microenvironment where the tumor is located, but also independently predict the overall survival and relapse-free survival of the patient. It is considered to be a better predictor of clinical outcome than the standard TNM staging (15, 16). Immune score has a good predictive value for the survival of patients with colon cancer (17), but the immune score about HCC were limited. According to the immune score, especially based on the distribution pattern of CD3^+^T and CD8^+^T lymphocytes, immune tumors are divided into three types: Immune-inflamed tumor; immune-excluded tumor; immune-desert tumor (18). Immuno-inflammatory tumors are in an activated or semi-activated state. Checkpoints inhibitors, such as programmed death receptor-1 (PD-1), programmed death receptor ligand-1 (PD-L1), are likely to exert anti-tumor effects in this immunophenotype; the immune-excluded tumor shows that there are a large number of immune cells around the tumor cells, but the immune cells cannot penetrate into the core of the tumor cells and are restricted to the peripheral matrix of the tumor cells. Due to the interaction and influence of many factors in the tumor cell matrix, it is difficult for checkpoints inhibitors to exert anti-tumor effects in this phenotype. After PD-1/PD-L1 inhibitor treatment, peripheral matrix-associated T cells have proliferation and activation, but there is no infiltration, and the clinical response is uncertain. Therefore, how to improve T cell migration is the rate-limiting step for this phenotype; Immune-desert tumor is characterized by the lack of T cells in the inner and outer matrix of tumor cells. PD-1/PD-L1 inhibitors have no any effect on this phenotype. How to induce more tumor-specific T cells is the Phenotypic restriction steps (19, 20).

Immune checkpoint inhibitor (ICI) is one of the most rapidly developed immunotherapies strategies of HCC in recent years. ICI can block tumor-induced immunosuppression, thereby enhancing the anti-tumor immune response. Immune checkpoint are inhibitory tumor immune receptors, which are located on the surface of activated T cells. After the immune checkpoint is combined with the tumor surface antigen, it can inhibit tumor immune response and promote tumor immune escape. And ICI mainly reactivate tumor-specific T cells and exert anti-tumor effects by inhibiting checkpoint-mediated signal transduction (21). ICI targets mainly include PD-1, PD-L1, and cytotoxic T lymphocyte antigen-4 (CTLA-4) (22). However, thorny issues such as super progress and immune tolerance also appear in the course of immunotherapy. Indeed, a lot of combinatory approaches are under investigation, including the combination of different ICI, the addition of ICI after resection or during loco-regional therapy, combination of anti-angiogenic drugs or molecular targeted drugs. Compared with single-agent therapy, ICI combination therapy also reflects better clinical efficacy and good safety (23). The emergence of ICI has brought new research directions to researchers, and we look forward to better development of immune checkpoint inhibitors in the future.

There are many reports on the mechanism of CXCL5 promoting cancer progression. The combination of CXCL5 and CXCR2 exerts a strong granulocyte chemotaxis and angiogenesis effect, and CXCL5/CXCR2 axis plays an important role in mediating the infiltration and metastasis of malignant tumors (8). Recently, the CXCL5/CXCR2 axis is sufficient to promote breast cancer colonization during bone metastasis (24). CXCL5 activated the PI3K-Akt and ERK1/2 signaling pathways in HCC cells and promoted proliferation, migration, and invasion (25). The expression level of CXCR2 in HCC was significantly higher than normal liver tissues, and the expression levels of CXCR2 mRNA and protein were associated with intrahepatic metastasis, portal vein tumor thrombus, and poor differentiation (26). A longitudinal study has indicated that the chronically increasing trend of CXCL5 were associated with the promotion of the progression of NAFLD to HCC in males (27). Xu’s study had shown that overexpression of CXCL5 in HCC cells has higher metastatic potential, which also demonstrated that CXCL5/CXCR2 and ERK1/2 highway may play an important role in the migration of HCC (28). Based on ONCOMINE, GEPIA, and cBioPortal databases, the expression levels of CXCL5 were correlated with different tumor stages and high transcriptional levels of CXCL5 may exhibit poorer overall survival in patients with HCC (29). Previous study had indicated that EGF/EGFR signaling pathway plays an important role in the production of CXCL5 in HCC, and then activates downstream signaling pathways, thus mediating inflammatory microenvironment, as well as cell proliferation, apoptosis, and metastasis, revealing the signaling pathway of CXCL5 in HCC. In this study, the expression of CXCL5 was correlated with the TMN stage of HCC and was verified in our hospital (30). By analyzing the correlation of CXCL5 with the proportion of TICs, there are significant correlation between CXCL5 expression and the proportion of NK cells activated, macrophages M0, mast cells resting, neutrophils. It was well-known that CXCL5 was crucial for the function activation of different cells, especially neutrophil. Up to now, there are many reports about the mechanism of CXCL5 promoting tumor progression. CXCL5 can also activate protein kinase B (PKB) and activator of transcription (STAT) signaling pathways and promote tumor angiogenesis (31). Also, CXCL5 promotes tumor angiogenesis, and new blood vessels act as tumor metastasis channels; CXCL5/CXCR2 can release matrix metalloproteinase-9 (MMP-9), destroys endothelial cells and matrix barrier, and promotes tumor metastasis (32). Recent reports have shown that Retinoic acid receptor-related orphan receptor (ROR)-α inhibits the proliferation, invasion, and migration of HCC MHCC97H *via* down-regulation of CXCL5 (33).

This study has several limitations. Firstly, although it has been verified by patients in our hospital, the main research of this study is bioinformatics analysis based on TCGA database, and functional experiments are necessary to reveal the predictive mechanisms of CXCL5. Secondly, confounding effects of treatment factors are different to control because of the lack of treatment information. considering that the main causes of HCC in different countries are different, proving our signature in more independent cohorts is necessary to expand our model to other populations, especially in patients with advanced stage of HCC (34).

In conclusion, we determined the TME-related genes in HCC using ESTIMATE algorithm in TCGA database. CXCL5 was a potential prognostic factor for HCC patients by intersection analysis of PPI network and univariate cox regression. Then, the expression of CXCL5 was significant corrected with TMN classification both in TCGA database and verification data. More interestingly, CXCL5 might be an indicator for the conversion of TME status from immune-dominant to metabolic-dominant. There was significant correction between CXCL5 expression and the proportion of NK cells activated, macrophages M0, Mast cells resting, Neutrophils. Our signature might reflect CXCL5 has potential to be a marker for HCC prognosis and correlating with immune infiltrates. However, validation of the signature in more independent cohorts from different country and functional experiments of the predictive genes are warranted.

## Data Availability Statement

The datasets presented in this study can be found in online repositories. The names of the repository/repositories and accession number(s) can be found below: https://portal.gdc.cancer.gov/.

## Ethics Statement

The studies involving human participants were reviewed and approved by the First Affiliated Hospital of Nanchang University (No. 2017-0106). Written informed consent was obtained from all participants. The patients/participants provided their written informed consent to participate in this study.

## Author Contributions

YN: study concept, design, and data analyzing. M-CJ and CL: experimental operation. QL: data collection. XZ: obtained funding and critically revised the manuscript. All authors contributed to the article and approved the submitted version.

## Funding

This study was supported by the National Natural Science Foundation of China (grant number: 81960120), “Gan-Po Talent 555” Project of Jiangxi Province [GCZ (2012)-1], and the Postgraduate Innovation Special Foundation of Jiangxi Province (YC2020-B046).

## Conflict of Interest

The authors declare that the research was conducted in the absence of any commercial or financial relationships that could be construed as a potential conflict of interest.
